# U‐curve relation between cholesterol and prior ischemic stroke

**DOI:** 10.1002/brb3.574

**Published:** 2016-09-22

**Authors:** Gerd Haga Bringeland, Aliona Nacu, Ulrike Waje‐Andreassen, Lars Thomassen, Halvor Naess

**Affiliations:** ^1^ Department of NeurologyHaukeland University HospitalBergenNorway; ^2^ Institute of Clinical MedicineUniversity of BergenBergenNorway; ^3^ Institute of Biological and Medical PsychologyUniversity of BergenBergenNorway; ^4^ Centre for Age‐Related MedicineStavanger University HospitalStavangerNorway

**Keywords:** cholesterol, ischemic stroke, reverse epidemiology, statin, stroke recurrence, vascular risk factors

## Abstract

**Objectives:**

Previous prospective studies on ischemic stroke patients have shown conflicting results concerning the association between cholesterol level and patient outcome. We aimed to investigate the relation between cholesterol level and prior ischemic stroke. We hypothesized that acute ischemic stroke patients with increased cholesterol on admission more frequently had experienced prior ischemic stroke.

**Methods:**

All consecutive patients with acute ischemic stroke (the index stroke) admitted to the Stroke Unit, Department of Neurology, Haukeland University Hospital between February 2006 and October 2013 were prospectively registered in The Bergen NORSTROKE Registry. On admission, cholesterol, low‐density lipoprotein, and high‐density lipoprotein levels were measured and prior ischemic stroke, risk factors, and medication were registered. Patients with prior versus no prior ischemic stroke were compared regarding risk factors, cholesterol levels, and use of statins on admission for the index stroke. Only patients with available cholesterol values measured on admission were included in the analyses.

**Results:**

Of the 2,514 included patients admitted with acute ischemic stroke, 429 (17%) patients had prior ischemic stroke. We found a U‐curve relationship between the relative frequency of prior ischemic stroke and cholesterol level. Lower frequency of prior ischemic stroke was associated with high cholesterol level on admission up to 5.5 mmol/L. For cholesterol levels higher than this, the opposite was true. These associations included all patients and statin‐naive patients. For patients using statin there was a declining relative frequency of prior ischemic stroke from low to high cholesterol levels.

**Conclusion:**

Our hypothesis was falsified. The association between lower cholesterol levels and higher frequency of prior ischemic stroke in patients with cholesterol <5.5 mmol/L cannot be solely an effect of aggressive statin treatment in patients with prior ischemic stroke, as the association pertained also to patients who did not use statin.

## 1 Introduction

High cholesterol level is a well‐known risk factor of atherosclerosis, which is one of the major causes of ischemic stroke (Amarenco, Labreuche, Lavallee, & Touboul, 2004). Previous prospective studies have shown conflicting results concerning the association between cholesterol level and outcome following ischemic stroke. Some studies have shown that lowering cholesterol with statin treatment has a positive effect on both functional and neurological outcome and decreases mortality and the risk of ischemic stroke recurrence (Amarenco et al., 2006; Athyros, Kakafika, Tziomalos, Papageorgiou, & Karagiannis, 2008; Navi & Segal, 2009; Ni Chroinin et al., 2013; Salat, Ribosa, Garcia‐Bonilla, & Montaner, 2009). Others have shown the opposite: that low cholesterol in patients with acute ischemic stroke is associated with increased stroke severity, poorer functional outcome, and increased mortality (Koton, Molshatzki, Bornstein, & Tanne, 2012; Markaki, Nilsson, Kostulas, & Sjostrand, 2014). In this study, we aimed to investigate the relation between cholesterol level on admission for acute ischemic stroke and prior ischemic stroke. We hypothesized that acute ischemic stroke patients with increased cholesterol on admission more frequently had experienced prior ischemic stroke.

## 2 Methods

All consecutive patients with acute ischemic stroke (the index stroke) admitted to the Stroke Unit, Department of Neurology, Haukeland University Hospital between February 2006 and October 2013 were prospectively registered in The Bergen NORSTROKE Registry. Ischemic stroke was defined in accordance with the Baltimore‐Washington Cooperative Young Stroke Study Criteria comprising neurological deficits lasting more than 24 hr because of ischemic lesions or transient ischemic attacks where CT or MRI showed infarctions related to the clinical findings (Johnson et al., 1995). Patients records regarding prior ischemic stroke defined according to the WHO criteria for ischemic stroke (Hatano, 1976) were consulted. In a few cases, patients or relatives reported strokes not registered in the patient records. These strokes were also included in the analyses.

On admission, a blood sample for measuring cholesterol, low‐density lipoprotein (LDL), and high‐density lipoprotein (HDL) was collected. Patients without available cholesterol levels measured on admission were not included in the analyses.

Risk factors were registered: Current smoking was defined as smoking at least one cigarette per day. Diabetes mellitus was considered present if the patient was on glucose‐lowering diet or medication. Hypertension, angina pectoris, myocardial infarction, and peripheral artery disease were considered present if diagnosed by a physician any time before stroke onset. Atrial fibrillation required ECG confirmation any time before stroke onset and was categorized as paroxysmal atrial fibrillation or chronic atrial fibrillation. Use of statins before the index ischemic stroke was registered.

Etiology was determined by the Trial of Org 10172 in Acute Stroke Treatment classification (TOAST) and classified as large‐artery atherosclerosis, cardioembolism, small vessel disease, other, and unknown (Adams et al., 1993).

Long‐term mortality data obtained from the official population registry on 1 January 2012 were available for all acute ischemic stroke patients registered in the Bergen NORSTROKE registry during the first 6 years of the inclusion period (between February 2006 and January 2012).

The study was approved by the local ethics committee (REK Vest).

### 2.1 Statistics

Chi‐square test was used for categorical variables. For continuous variables, we used Students *t* test and Mann–Whitney test as appropriate. Stepwise forward logistic regression analyses were performed based on variables in Table 1, starting with one significant variable (age) and then stepwise adding one additional significant variable. Because of interaction with cholesterol, LDL and HDL were not included in the model and neither was TOAST classified. Cox regression analyses were performed on the subgroup of patients where long‐term mortality data were available. In Cox regression analyses, only sex, age, and cholesterol (as continuous variable, measured in mmol/L) were included. Lowess smoother curves were obtained as shown. STATA 13.1 (StataCorp, College Station, TX) was used for the analyses.

**Table 1:** Demographics of patients with prior and no prior ischemic stroke

	Prior ischemic stroke	No prior ischemic stroke	*p*‐value
Age (*SD*)	75.1 (12.6)	70.2 (15.0)	<.001
Male	171 (40)	880 (43)	.27
Female	258 (60)	1,179 (57)	
Prior statin	206 (48)	437 (21)	<.001
Cholesterol in all (*SD*)	4.9 (1.4)	5.4 (1.3)	<.001
Cholesterol in statin‐naive (*SD*)	5.5 (1.4)	5.7 (1.2)	.04
LDL (*SD*)	3.1 (2.3)	3.5 (1.1)	<.001
HDL (*SD*)	1.4 (.4)	1.5 (.5)	.007
Prior myocardial infarction	84 (20)	272 (13)	<.001
Hypertension	281 (66)	1,017 (49)	<.001
Diabetes mellitus	79 (19)	270 (13)	.002
Paroxysmal atrial fibrillation	63 (15)	167 (8)	<.001
Chronic atrial fibrillation	59 (14)	171 (8)	<.001
TOAST classification			
Large‐artery atherosclerosis	66 (15)	232 (11)	.02
Cardiac embolism	150 (35)	669 (32)	.32
Small vessel disease	48 (11)	265 (13)	.34
Unknown	158 (37)	823 (40)	.23

## 3 Results

In total, 2,514 (93%) of the 2,697 patients admitted with acute ischemic stroke (the index stroke) during the inclusion period had available cholesterol values and were included in the study: 429 (17%) with prior ischemic stroke and 2,059 (83%) without prior ischemic stroke. Table 1 shows demographic data, risk factors and laboratory parameters of patients with and without prior ischemic stroke. Patients with prior ischemic stroke had lower cholesterol and lower LDL levels (both *p* < .001). Risk factors such as hypertension, diabetes mellitus, and paroxysmal or chronic atrial fibrillation were significantly more frequent among patients with prior ischemic stroke. Only large‐artery atherosclerosis was associated with prior ischemic stroke based on the TOAST classification of the index stroke.

**Figure 1:**
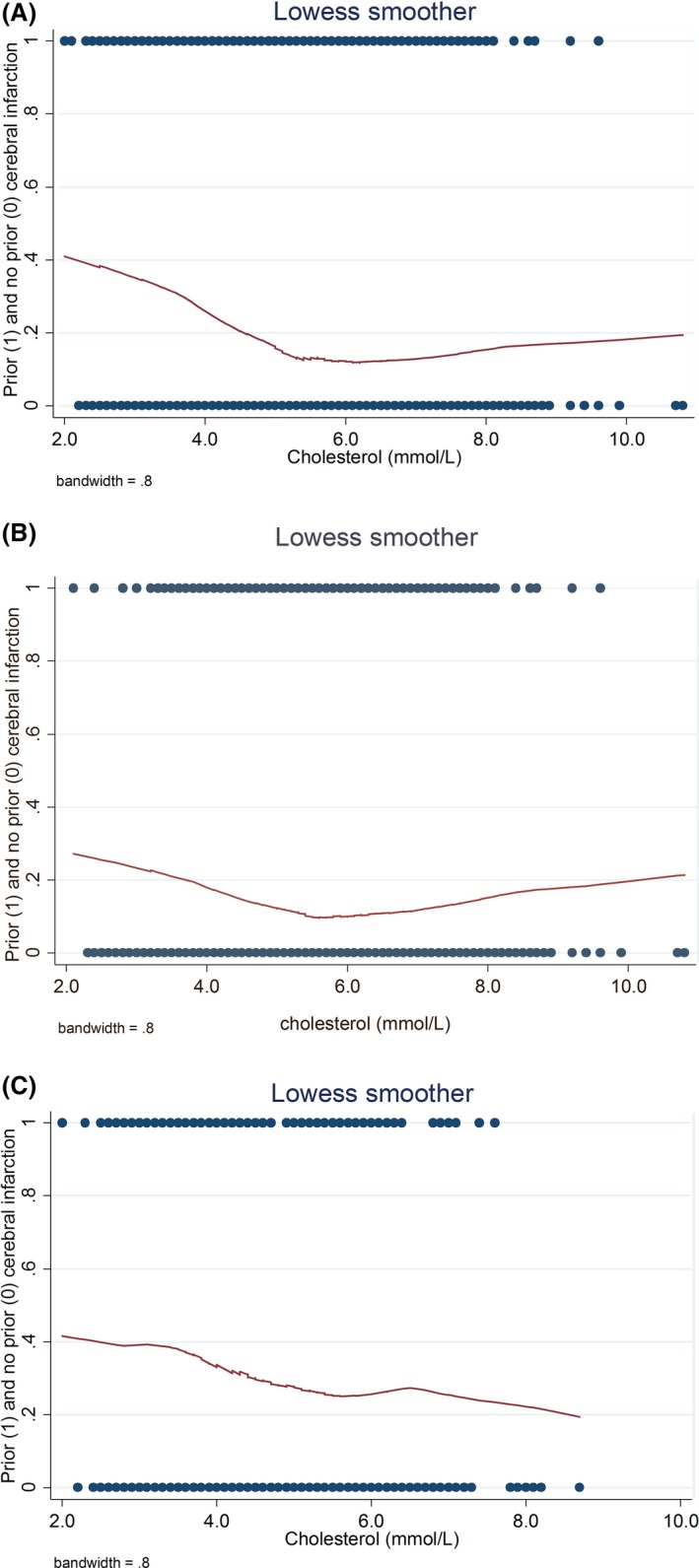
Lowess smoother showing prior versus no prior ischemic stroke according to cholesterol level on admission in (A) all, (B) statin‐naive, and (C) statin‐using acute ischemic stroke patients

Figure 1 shows that there was a U‐curve relationship between the relative frequency of prior ischemic stroke and cholesterol level. Lower frequency of prior ischemic stroke was associated with increasing cholesterol level on admission up to 5.5 mmol/L. For cholesterol levels higher than this, the opposite was true. This pertained to all patients (Fig. 1A) and to patients who did not use statin prior to admission (Fig. 1B). For patients using statin, there was declining relative frequency of prior ischemic stroke from low to high cholesterol levels (correlation = .16, *p* < .001; Fig. 1C).

Table 2 shows that, consistent with our hypothesis, there was a positive relationship between the relative frequency of prior ischemic stroke and cholesterol level for cholesterol >5.5 mmol/L after adjusting for confounders (odds ratio [OR] = 1.3, *p* = .02). In contrast there was an inverse relationship between the relative frequency of prior ischemic stroke and cholesterol level for cholesterol <5.5 mmol/L after adjusting for confounders (OR = .6, *p* < .001). Patients with prior ischemic stroke more often used statins than patients with no prior ischemic stroke, both in the group with cholesterol level <5.5 mmol/L (OR = 2.6, *p* = <.001) and >5.5 mmol/L (OR = 3.1, *p* = <.001). Sex and age were not associated with prior ischemic stroke.

**Table 2:** Logistic regression of prior versus no prior ischemic stroke in patients with acute ischemic stroke and cholesterol >5.5 and <5.5 mmol/L on admission

	>5.5 mmol/L			<5.5 mmol/L		
	Odds ratio	Confidence interval	*p*‐Value	Odds ratio	Confidence interval	*p*‐Value
Age	1.03	1.01–1.04	.002	1.03	1.01–1.04	<.001
Sex	1.3	0.8–1.9	.27	0.94	0.7–1.3	.67
Cholesterol	1.3	1.04–1.6	.02	0.6	0.5–.8	<.001
Prior statin	3.1	1.8–5.3	<.001	2.6	1.9–3.5	<.001
Prior myocardial infarction				0.6	0.4–.9	.005

Table 3 shows the differences between the patients who used statins before the index stroke and the patients who were statin‐naive. Prior to the index stroke, 22% of female and 29% of male patients used statins (*p* < .001). Statin users were older than patients not using statins (age 73.7 years vs. 70 years, *p* < .001). Prior myocardial infarction was more common among the patients using statins (34% vs. 7.5%, *p* < .001), as was hypertension (69% vs. 47%, *p* < .001).

**Table 3:** Demographics of patients with and without statin treatment before the index ischemic stroke

	Prior statin	No statin	*p*‐value
Age (*SD*)	73.7 (11.3)	70.0 (15.8)	<.001
Female (%)	256 (22)	888 (78)	<.001
Male	450 (29)	1,103 (71)	
Prior myocardial infarction	238 (34)	149 (7.5)	<.001
Hypertension	485 (69)	933 (47)	<.001

Long‐term mortality data were available on all patients included during the first 6 years of the inclusion period, in total 1,867 patients. By the end of the observation period on 1 January 2012, 437 (23%) of these were dead. Mean observation time (until death or end of the observation period) was 2.2 years (*SD* 1.7 years). Cox regression analysis showed that long‐term mortality was associated with age (hazard ratio [HR] = 1.1, *p* < .001) and low cholesterol (HR = .86, *p* = .003), but not sex (HR = 1.0, *p* = .7) in patients with no prior cerebral infarction.

## 4 Discussion

Our study did not confirm that acute ischemic stroke patients with increased cholesterol level on admission more frequently had experienced prior ischemic stroke. Instead, we found a U‐curve relationship where lower frequency of prior ischemic stroke was associated with high cholesterol level on admission up to 5.5 mmol/L, whereas the opposite relationship held for patients with cholesterol >5.5 mmol/L. Thus, our hypothesis was falsified. This U‐curve relationship was found among all patients and patients who were statin‐naive on admission. Thus, the low cholesterol levels cannot be solely explained by an effect of statin treatment after the first ischemic stroke.

For patients who used statins prior to the index stroke, the frequency of prior ischemic stroke decreased with increasing cholesterol. This association could be influenced by possibly more aggressive cholesterol‐lowering therapy in patients with prior ischemic stroke.

The mechanisms behind the associations between cholesterol level, statin treatment and ischemic stroke appear to be diverse. The nonlinear U‐curve relationship between cholesterol levels and previous ischemic stroke in our data could be due to separate mechanisms for the association between risk of stroke recurrence and cholesterol level for the different etiological subtypes of ischemic stroke. The association between cholesterol level and recurrence of ischemic stroke of different etiological subtypes should be further investigated in future prospective studies.

Studies on patients with coronary heart disease have mainly shown a clear reduction in cardiovascular events proportional to the reduction in LDL‐cholesterol with statin treatment (Cannon et al., 2015; Packard, 2015). Most previous studies of the association between cholesterol levels and outcome after cerebral ischemic stroke have considered the effect of statin treatment rather than cholesterol levels per se. Cholesterol levels have been shown to be only weakly associated with ischemic stroke (Fonseca, Franca, Povoa, & Izar, 2010). Statins seem to reduce vascular risk beyond that expected from cholesterol reduction alone and decrease stroke incidence also in populations with a normal baseline cholesterol concentration. Based on this, some authors have proposed statins to have cholesterol‐independent (also referred to as pleiotropic) effects such as improving endothelial function, enhancing the stability of atherosclerotic plaques, decreasing oxidative stress and inflammation, and inhibiting the thrombogenic response (Robinson, Smith, Maheshwari, & Schrott, 2005; Vaughan, Delanty, & Basson, 2001).

In some studies, low cholesterol has been associated with higher rates of stroke risk factors, and poorer outcome both in patients with and without statin treatment (Koton et al., 2012; Markaki et al., 2014). This resembles a so called reverse epidemiology phenomenon, previously reported in many other medical conditions, which refers to the opposite effect between certain risk factors and morbidity or mortality in some chronic diseases in comparison to the general population (Beltowski, 2014).

One of the strengths of this study is the large number of patients included in a single center based on a predefined protocol. A weakness is that registration of prior ischemic stroke was partly based on patient and relatives’ recall, but this pertained only for a minority of the patients as most prior strokes were registered in the patient records. Ideally, the study should have been performed prospectively. However, this demands a higher number of patients and longer observation time to obtain enough events of recurrent stroke. The retrospective design as to registration of prior cerebral infarction could cause selective inclusion of patients: If high cholesterol levels would increase mortality, shorter survival time would reduce the chance of patients with prior ischemic stroke and high cholesterol levels to get a recurrent stroke and get included in this study. However, Cox regression analysis based on prospective registration of long‐term mortality in a subgroup comprising a majority of the included patients showed the opposite to be the case: even mortality was associated with low cholesterol on admission. This supports that our findings are valid, but further investigation in future prospective studies is needed.

In conclusion, prior ischemic stroke had a U‐shaped relation to cholesterol level in acute ischemic stroke in statin‐naive patients. This suggests that cholesterol may be a factor contributing in different mechanisms associated with acute ischemic stroke.

## Conflict of Interests

The authors declare that they have no conflict of interest.
